# The Influence of Knee Proprioception and Strength on Lower-Limb Functional Symmetry in Healthy Adults

**DOI:** 10.3390/muscles4010003

**Published:** 2025-01-21

**Authors:** Joffrey Drigny, Marine Rolland, Antoine Gauthier

**Affiliations:** 1 Department of Sports Medicine and Physical Medicine and Rehabilitation, CHU de Caen Normandie, Normandie Univ, 14000 Caen, France; 2 INSERM, COMETE, Université de Caen Normandie, 14000 Caen, France

**Keywords:** functional test, hop testing, movement system, joint position sense

## Abstract

This study examined the association between knee proprioception, strength, and functional symmetry in healthy individuals using isokinetic strength tests, proprioception assessments, and hop tests. Twenty young, healthy adults (mean age 26.5 ± 4.1 years, 85% right-limb dominant) participated. Knee extensor and flexor strength were measured at 60°·s^−1^ and 240°·s^−1^. Proprioception was assessed by measuring passive joint position sense (JPS1: position recognition; JPS2: repositioning) and kinesthesia (threshold to detection of passive motion, TTDPM). Functional performance was evaluated using the single-leg hop test (SLH), triple-hop test (TH), and crossover hop test (COH). Symmetry was calculated using the limb symmetry index (LSI) as the ratio of non-dominant to dominant limb values. The results showed that THT (*p* = 0.011) and COH (*p* = 0.032) performance was superior on the dominant limb. A correlation analysis revealed strong positive correlations between hop test distances and knee extensor strength (r = 0.56–0.70, *p* < 0.001). JPS symmetry was negatively correlated with hop test symmetry (JPS1: SLH, r = −0.53; THT, r = −0.49; COH, r = −0.70). The participants with poorer position sense on the non-dominant leg were 2.7 times more likely to show LSI < 90% (*p* = 0.035). In conclusion, proprioception—particularly joint position sense—is associated with functional symmetry during dynamic tasks, highlighting the importance of proprioceptive assessments in rehabilitation and injury prevention.

## 1. Introduction

Knee function plays a critical role in preserving mobility, stability, and overall performance in both healthy adults and athletes [1]. Adequate knee function is essential for optimizing performance in both sports and daily activities, while also reducing the likelihood of long-term musculoskeletal problems and enhancing quality of life. Several factors contribute to knee function, including muscle strength and proprioception. Lower limb strength, particularly in the quadriceps and hamstrings, is crucial for maintaining knee stability. A well-balanced strength between these muscle groups not only improves athletic performance by supporting explosive movements and endurance but also lowers the risk of knee injuries in both healthy individuals and athletes [2]. Knee proprioception, which refers to the body’s ability to sense joint position and movement, is essential for balance, coordination, and efficient movement, playing a key role in both athletic performance and everyday activities [3]. However, the specific contributions of these factors to functional knee tasks remain not fully understood.

The single-leg hop for distance tests (hop tests) are common and reliable evaluations to assess the functional status of athletes [4]. Hop tests have been described to assess overall balance ability, which is related to different physical capacities such as strength, neuromuscular control, and proprioception [5]. These tests have been largely used to assess lower limb performance and between-limb symmetry as decision criteria for return to sport after lower limb injury, especially after anterior cruciate ligament reconstruction (ACLR) [4,6]. A recent systematic review with meta-analysis has found higher odds of return to sport, better self-reported symptoms and function, and reduced odds of knee osteoarthritis at a minimum of 5 years after ACLR [7]. Also, hop test symmetry has potential use in injury prevention to identify athletes at risk for lower back or lower limb injuries [8]. Thus, a better understanding of the factors contributing to hop performance would help clinicians identify potential deficiencies.

In addition to functional tests, the isokinetic testing of the knee joint is the gold standard for measuring knee flexor/extensor strength and has been validated for assessing muscular symmetry in injured and healthy athletes [9,10]. Other types of tests, such as surface electromyography (sEMG), imaging, and various forms of dynamometry, could serve as complementary methods for exploring muscle function [11]. Isokinetic testing has also demonstrated promising psychometric properties for assessing knee joint proprioception, particularly by measuring kinesthesia and joint position sense [12]. Previous studies have investigated the association between hop test performance and knee strength and found that decreased muscle strength, passive muscle stiffness, and muscle activation of the quadriceps and hamstrings were associated with poorer single-leg hop performance [4,13]. Gokeler et al. performed a systematic review assessing the clinical relevance of proprioceptive deficits after ACL injury and concluded that the correlation between hop tests and proprioception cannot be established [14]. However, specific knowledge is lacking about the factors associated with hop test symmetry, which is most commonly used in clinical practice [15]. Moreover, most of the articles have included injured participants, and better identification of the factors influencing hop test symmetry in healthy individuals would help in understanding the underlying mechanisms contributing to functional symmetry. Indeed, a systematic review exploring the impact of limb dominance during functional tasks has described a noticeable trend towards increased distance on the dominant leg relative to the non-dominant leg during hopping tasks, but the impact of limb dominance on hop test performance and symmetry is still uncertain [16].

This study aimed to evaluate the association between functional single-leg hop testing and isokinetic assessment of strength and proprioception in both performance and limb symmetry in healthy athletes. We hypothesized that hop test performance would correlate with knee strength, while asymmetry would be influenced by both strength and proprioception.

## 2. Materials and Methods

### 2.1. Design

This cross-sectional study was conducted among healthy individuals in the Sports Medicine Department of a single tertiary university hospital in Caen, France. All the subjects provided written informed consent in accordance with the Declaration of Helsinki. This study was approved by an independent ethics committee for research (Comité de Protection des Personnes, Ile de France XI, 19046-52621) and registered with Clinical Trials (NCT04885725). The study population consisted of the control group from a larger research project investigating the impact of ACLR on knee proprioception [17], with the procedures and analyses presented in this paper specifically designed for the purposes of the present study.

### 2.2. Participants

The participants were recruited from the university community and included adult amateur and/or recreational athletes aged 18–45 years. An exponential discriminative snowball sampling method was used to achieve the target sample size, and all newly referred individuals were screened against the eligibility criteria. The exclusion criteria were current or recent lower limb injury causing time loss from sports participation and a history of severe knee injury (e.g., severe ligament tear or knee fracture). Additionally, participants with medical conditions or medications affecting balance or coordination were excluded.

### 2.3. Procedures

**Figure 1:**
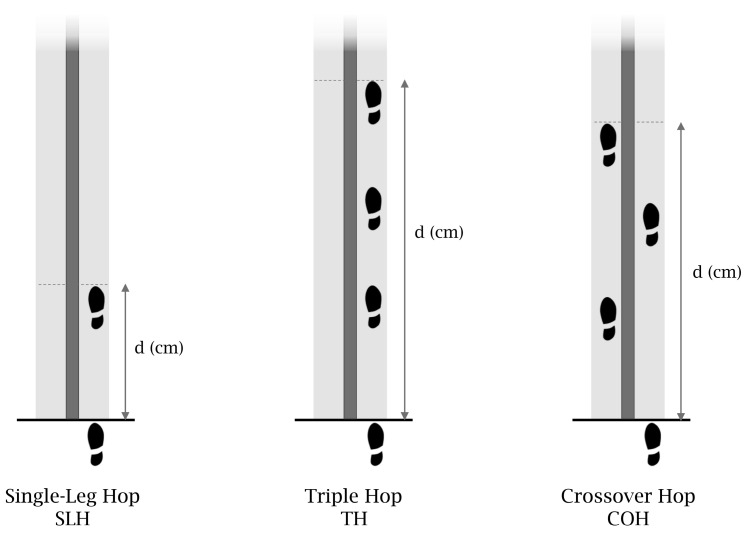
Diagrammatic representation of the 3 hop tests used to determine functional performance and symmetry. Legend: d: distance.

The participants had one visit and underwent the functional tests and the isokinetic tests as described below. For all the tests, the dominant limb was considered as the preferred limb for kicking a ball and, both the dominant and non-dominant legs were tested in a randomized order. Hop tests are single-leg horizontal tests for distance, and we used the single hop test (SLH), triple hop test (TH), and crossover hop test (COH) for distance [4]. A 6 m long, 15 cm wide line was marked on the floor and a standard tape measure was fixed to the ground, perpendicular to the starting line of the test (Figure 1).

The subjects had familiarization trials and, then, performed 2 trials being measured (landing on the same leg and maintaining stability for three seconds), and the best trial was collected for performance. Performance (distance, d) was measured and distance normalized by body height was calculated as d_norm_= d (cm)/height (cm) [1]. The limb symmetry index (LSI) was calculated as non-dominant/dominant * 100% and an LSI > 90% was considered adequate for limb symmetry in healthy individuals [18].

The knee isokinetic test was performed using an isokinetic Con-Trex^®^ isokinetic dynamometer (Con-Trex MJ; CMV AG, Dübendorf, Switzerland) in the sitting position. The protocol is similar to the one presented in research assessing proprioception in ACLR patients [17]. All the participants performed a familiarization set of submaximal repetitions for each condition. Proprioception was assessed prior to the maximal strength tests to avoid any influence on proprioceptive performance.

**Figure 2:**
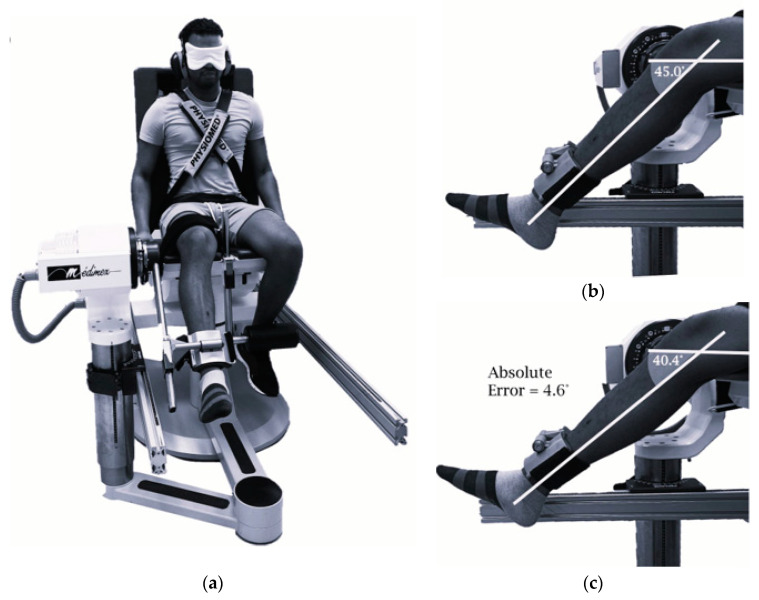
(**a**) Participant position and testing setup for knee joint position sense (JPS) measurement using the isokinetic dynamometer with (**b**) true target angle and (**c**) reproduced angle.

For proprioception, the awareness of joint position was measured with the joint position sense (JPS) and kinesthesia was measured with the threshold to detection of a passive motion (TTDPM) [12]. For both evaluations, all the participants wore eye masks during the testing to prevent visual input (Figure 2). Following familiarization trials, the participants completed two measured trials, with the best performance recorded for analysis.

To assess JPS, the knee was positioned at a target angle of 45° flexion. After returning the limb to 90° flexion, the participants reproduced the target angle. For JPS1, the limb was passively moved at 2°·s^−1^, and the participants pressed a stop switch upon reaching the angle. For JPS2, the participants controlled the limb’s movement with a remote to reproduce the angle. Absolute error (AE) was the difference between the target and reproduced angles, with higher values indicating poorer proprioceptive accuracy [19]. TTDPM was measured by passively extending the knee from 90° flexion at 0.2°·s^−1^. The participants pressed a stop switch when they detected movement. AE was calculated as the difference between the initial and final positions, where higher values indicated poorer kinesthetic sense. For JPS1, JPS2, and TTDPM, the absolute difference between limbs was calculated as AE_diff_ = AE_non-dom_ − AE_dom_.

For strength testing, the participants warmed up with 2 sets of 10–15 submaximal contractions at 180–240°·s^−1^. Data were collected during 4 maximal concentric repetitions of knee extensors/flexors at 60°·s^−1^ and 240°·s^−1^, with verbal encouragement. The limb symmetry index (LSI) was calculated as non-dominant/dominant *100%, and > 90% was used to determine readiness to return to sports [6] and was considered as adequate in elite athletes [20].

### 2.4. Statistical Analysis

The mean and standard deviation (mean ± SD) were calculated for continuous variables. The Shapiro–Wilk test was used to test the normality of the data. A paired *t*-test was used to assess the difference between the dominant and non-dominant limbs. The Pearson correlation coefficient was used to assess the association between the isokinetic and functional testing performances and/or symmetry indexes. The correlation coefficient (r) was categorized as follows: 0–0.1 (trivial), 0.1–0.3 (weak), 0.3–0.5 (moderate), and >0.5 (strong) [21]. To assess the predictive accuracy of the isokinetic parameters associated with functional symmetry (LSI > 90%), we used the receiver operating characteristic (ROC) analysis and Fisher’s exact test of independence. For sample size calculation and using an estimated *r* = 0.6 corresponding to the correlation between the distance hopped and knee-extension peak torque (*r* = 0.63 from English et al.) [22], a minimum sample size of 19 was calculated (*α* = 5%, *β* = 20%). Statistical analyses were performed using the SPSS software (Version 25.0; IBM Corp., Armonk, NY, USA).

## 3. Results

Twenty young healthy adults (men = 10 and women = 10; age = 26.5 ± 4.1 y; height = 174.2 ± 8.4 cm; body mass = 67.2 ± 10.4 kg; right leg dominant = 85%) volunteered to participate in the study and were included. The median Tegner score was 6 (Q1–Q3: 6–7), and 60% of the participants engaged in competitive sports. Both the dominant and non-dominant legs were tested for all the participants, and no adverse event was noted or reported during and after the experiments.

The performances for functional tests and isokinetic strength and proprioception tests, with bilateral difference analysis, are presented in Table 1. The performances on the TH (t = 2.83, *p* = 0.011) and COH tests (t = 2.31, *p* = 0.032) were significantly better on the dominant limb compared to the non-dominant limb. For kinesthesia, the TTDPM was significantly higher on the non-dominant limb (t = −2.57, *p* = 0.019). For strength, only extensors at 60°·s^−1^ (t = 3.07, *p* = 0.006) were weaker on the non-dominant limb.

The correlation analysis results are presented in Table 2(a,b).

Regarding the performance, the extensor peak torques at 60 and 240°·s^−1^ (*p* < 0.001) and flexor peak torque at 240°·s^−1^ (*p* < 0.050) were associated with better performances on the hop tests. Regarding, the limb symmetry AE_diff_ for the JPS1 was significantly negatively correlated with all the LSI_s_ of the hop tests (*p* < 0.001), and AE_diff_ for the JPS2 was significantly negatively correlated with the LSI on the SLH (*p* < 0.050) and COH tests (*p* = 0.050). The ROC curve analyses showed that the JPS1 had accurate predictive ability in discriminating individuals with LSI < 90% for at least one hop test (AUC = 0.879, CI95%: 0.726–1.000, *p* = 0.004). Fisher analysis indicated a significantly higher rate of individuals with LSI < 90% with a negative JPS1 AE_diff_ compared to individuals with a positive JPS1 AE_diff_ (80% vs. 30%, *p* = 0.035), so individuals having a worse JPS1 on the non-dominant leg compared to the dominant leg had a 2.7-fold higher risk (risk ratio = 2.667) of having LSI < 90% for at least one hop test.

## 4. Discussion

This study aimed to evaluate the association between knee functional tests and isokinetic knee strength and proprioception performance and symmetry. The main results are that performances on the hop tests were associated with higher knee muscle strength, whereas functional limb symmetry on the hop tests was associated with knee-joint proprioception, especially position sense.

These results demonstrated that performances on the TH and COH tests were significantly greater on the dominant limb compared to the non-dominant limbs, which supports that limb dominance impacts functional performances during single-leg hopping tasks [16,23]. Regarding the factors that influenced the functional performance, our results have found significant strong correlations between the knee extensor muscle strength (60 and 240°·s^−1^) and hop distances. Previous studies have shown similar findings in healthy individuals [22,24] and after ACLR [25]. The moderate but significant correlations between knee flexor muscle strength (240°·s^−1^) and hop distances may be explained by the important role of the hamstring muscle in the propulsive phase of the single leg hop [26]. The hamstrings contribute to generating greater hip extension strength during a forward hop task, playing a key role in propulsion to produce forward momentum. Additionally, enhancing hamstring muscle activity to achieve a balanced co-contraction with the quadriceps is crucial for effective landing, particularly in unstable conditions, contributing to regulating ACL loading in healthy individuals [27,28]. Isokinetic knee extension and flexion strength testing, alongside hop tests, serve as valuable tools for assessing bilateral knee strength and function, particularly in the context of guiding athletes’ return to sport. Gaining a deeper understanding of the relationship between these measures can empower clinicians, especially those without access to isokinetic testing equipment, to make evidence-based decisions regarding safe return-to-sport timelines, particularly following ACLR.

The most significant finding that emerged from this study was that the symmetry of position sense was the most significant parameter associated with functional symmetry on the hop tests. Our results have shown significant moderate-to-strong correlations between the JPS AE_diff_ and the LSI during the hop tests. Thus, the limb symmetry on the hop tests was more likely to be due to the proprioceptive sense of the knee joint than the knee flexor/extensor strength limb symmetry. In addition, when individuals had worse position sense on the non-dominant leg, they were 2.7-fold more likely to have LSI < 90% on the hop tests. Thus, the knee position sense of the non-dominant limb could be an important factor contributing to functional symmetry during unilateral hopping tasks. Interestingly, most of the participants (85%) were right-limb dominant, and previous findings have demonstrated the dominance of the right hemisphere for proprioception in the lower limbs [29,30]. Indeed, it has been supported that better proprioceptive acuity for the non-dominant limb compared with the dominant knee, especially among right-limb dominant individuals, indicates that hemisphere lateralization may have meaningful implications for motor control [31]. Galamb et al. demonstrated that right-side-dominant individuals perform knee joint target-matching tasks more accurately with the non-dominant leg than left-side-dominant individuals [32]. The present study did not demonstrate a significant difference in knee position sense between the dominant and non-dominant limbs, but better position sense on the non-dominant limb being associated with better functional symmetry during hop tests supports the fact that knee position sense could have a meaningful impact on functional motor tasks. The hop tests entail jumping and landing maneuvers for which evidence suggests the important role of proprioception and that improved knee proprioception is related to better functional joint stability, especially in landing techniques [33]. The best correlation was found between position sense symmetry and COH symmetry, and COH requires not only forward landing-jumping, but also implies that mediolateral component and knee stability are crucial to control frontal plane knee movements and loads [4]. The results revealed poorer kinesthetic accuracy, as evidenced by greater errors in detecting joint movement, a finding previously discussed [17]. Interestingly, Strong et al. (2023) reported better knee position sense acuity in the non-dominant limb among right-limb-dominant individuals, potentially due to lateralization and right hemisphere processing [31]. However, joint position and movement accuracy may not align, as the proprioceptive identification of joint position is often preferred over kinesthetic movement reproduction [34]. Proske and Gandevia (2009) suggest that these differences arise from distinct central and peripheral pathways for position and movement sense [35]. Kinesthesia, relying more on movement cues with lower memory demands, may involve less interhemispheric integration [12]. Furthermore, kinesthesia’s link to force perception is evident in strength training adaptations, where the dominant limb shows greater force reproduction improvements [36]. Further studies are needed for better comprehension of the specific role of limb preference on the distinct aspects of proprioception.

Although proprioceptive signals arise from several mechanoreceptors, it is accepted that muscle spindles are the main source of information for position sense [37]. In the muscle–tendon unit, muscle spindle sensory information encodes muscle stretch and length [38], which is distinct from that provided by Golgi tendon organs, which respond to changes in muscle load [39] but are relatively insensitive to passive stretch [40]. Thus, the main sensory input influencing passive JPS ability arises from muscle spindles. Also, the position of testing at 45° of knee flexion used as the target position in the present study is located in the mid-range of knee joint angles and corresponds to the position where adjustments by the muscle mechanoreceptors play a dominant role in detecting knee joint position [41]. Muscle spindles are involved in plyometric tasks, such as hopping, and especially through a stretch reflex [42]. Muscle spindles are crucial in encoding joint position and velocity, as well as muscle force, stiffness, velocity, and acceleration [43]. The significance of muscle spindles in maintaining locomotor function is especially clear when external disturbances occur, as their feedback is tightly linked to the modularity of locomotor control [44]. Indeed, feedback from muscle spindles helps fine-tune motor output in response to environmental demands, and without this feedback, the system remains in a constant state of disruption, struggling to adapt to changes [45]. It has been suggested that better joint proprioception related to increased sensitivity of the muscle spindle could be the main mechanism for the improvement of jumping and sprinting capacities of individuals involved in plyometric training [46]. This supports the association between joint position symmetry and functional symmetry, but the specific mediating role of muscle spindles requires further exploration.

Providing data documenting instrumental measurement of the knee joint associated with knee functional symmetry would help clinicians and researchers. So far, limb asymmetry on hop tests has been mostly considered as low performance on the non-dominant or injured leg and interpreted as a deficit in strength on the involved leg [47]. However, it has been suggested that other factors could explain functional symmetry on hop tests [4] and the present results support this hypothesis. These results will help for better analyzing and interpreting patients’ results on hop tests for both the primary and secondary prevention of knee injuries. Exploring proprioception through passive reproduction tasks is harmless and can be implemented early after injury or surgery. This study provides evidence of its association with functional tasks, supporting its use in aiding recovery across different stages. Given the association between position sense and hop test symmetry found in this study, proprioceptive training could play a key role in improving movement symmetry and overall performance. Enhancing proprioceptive feedback may help prevent injuries by fostering better movement patterns, particularly in both clinical and sports settings [3]. Finally, these results open up numerous perspectives for future research. Comparing the impact of knee proprioception in both right- and left-dominant-limb individuals on motor function could explore the impact of the right hemisphere dominance for proprioception on knee function and studying athletes from asymmetric sports. Also, other parameters could be analyzed for assessing landing kinematics such as knee-flexion angles or valgus/varus angles, which may increase the risk of knee injury in healthy individuals [48]. Finally, further studies should test the impact of knee dominance on proprioceptive sense while assessing the meaningful contributors to functional symmetry during hopping and landing tasks after ACLR.

These results are presented with caution, as there are several limitations to discuss. The participants were not sampled based on the type of sport they practiced, and leg preference could vary among athletes participating in sports with or without asymmetric kinetic patterns. Additionally, a few participants engaged in knee-strenuous sports. This study is a methodological technical report conducted on a limited sample to assess the validity of hop test symmetry, with a focus on young adults, which may reduce the generalizability of the findings to the broader healthy population. Further studies with larger samples are warranted to evaluate the specific impacts of age, gender, and the sport practiced. Research should also investigate the effects of proprioceptive training on functional knee symmetry in both healthy and injured athletes. Moreover, with the advancement of artificial intelligence and machine learning methods, future studies should investigate the role of proprioception and its influence on functional symmetry, exploring patterns and regularities in this area, particularly in the contexts of physical and rehabilitation medicine and sports sciences [49,50]. Finally, not only the knee, but also the hip and ankle joints are involved during hopping tasks, and further studies should include the testing of multiple joints. However, knee joint work contribution is predominant in the landing phase of horizontal hops [51], which is important for identifying individuals at greater risk of injury, probably because it more accurately reflects the mechanism of the injury [52].

## 5. Conclusions

The results from this study indicate that hop test symmetry is associated with proprioceptive joint position sense, whereas hop test performance is mostly correlated with knee extensor strength, and to a lesser extent flexor strength. Thus, proprioception is more likely to influence functional symmetry between the dominant and non-dominant legs during single-leg hopping tasks, whereas knee muscle strength is more likely to influence hop test performance.

These findings will help to better interpret asymmetry in hop tests with clinical implications in both the primary and secondary prevention of musculoskeletal injury in sports.

## Figures and Tables

**Table 1:** Performances on hop tests and isokinetic strength and proprioception tests with side-to-side difference analysis (n = 20).

			Performance		Symmetry
			Dominant Limb	Non-Dominant Limb	Diff (°) or LSI (%)
Isokinetic testing	Proprioception/ Kinesthesia	JPS 1 (°)	2.66 (2.2)	2.35 (2.49)	−0.31 (3.78)
		JPS 2 (°)	2.10 (1.65)	2.51 (2.2)	0.41 (3.25)
		TTDPM (°)	2.08 (1.2)	3.27 (2.85) *	1.20 (2.08)
	Strength	ext 60°·s^−1^ (N·m·kg^−1^)	2.42 (0.56)	2.31 (0.63) *	89.41% (14.23%)
		flex 60°·s^−1^ (N·m·kg^−1^)	1.38 (0.31)	1.42 (0.41)	94.50% (25.66%)
		ext 240°·s^−1^ (N·m·kg^−1^)	1.61 (0.46)	1.50 (0.37)	95.81% (18.32%)
		flex 240°·s^−1^ (N·m·kg^−1^)	1.15 (0.39)	1.06 (0.28)	98.21% (25.14%)
Functional testing	Hop tests	SLH (dist/height, m)	0.85 (0.13)	0.83 (0.12)	97.50% (6.94%)
		TH (dist/height, m)	2.51 (0.34)	2.39 (0.35) *	95.31% (7.45%)
		COH (dist/height, m)	2.40 (0.31)	2.28 (0.35) *	95.23% (9.63%)

Note: JPS: joint position sense; TTDPM: threshold to detection of a passive motion; ext: knee extensor muscles; flex: knee flexor muscles; SLH: single hop test; TH: triple hop test; COH: crossover hop test. * indicates a significant difference between the dominant and non-dominant limbs with a *p*-value < 0.05.

**Table 2:** Analysis of correlations between (a) the performances on hop tests and isokinetic strength and proprioception tests and (b) the symmetry on hop tests and isokinetic strength and proprioception tests (n = 20, 40 legs).

(a) Performance				
		SLH (Dist/Height, m)	TH (Dist/Height, m)	COH (Dist/Height, m)
**Proprioception/Kinesthesia**				
JPS 1 (°)	Pearson’s r	−0.07	0.00	0.18
	*p*-value	0.664	0.985	0.254
JPS 2 (°)	Pearson’s r	0.07	0.11	0.00
	*p*-value	0.682	0.510	0.990
TTDPM (°)	Pearson’s r	−0.11	−0.26	−0.23
	*p*-value	0.515	0.104	0.162
**Strength**				
ext 60°·s^−1^ (N·m·kg^−1^)	Pearson’s r	0.65 **	0.58 **	0.56 **
	*p*-value	<0.001	<0.001	<0.001
flex 60°·s^−1^ (N·m·kg^−1^)	Pearson’s r	0.31	0.19	0.22
	*p*-value	0.052	0.229	0.169
ext 240°·s^−1^ (N·m·kg^−1^)	Pearson’s r	0.70 **	0.69 **	0.66 **
	*p*-value	<0.001	<0.001	<0.001
flex 240°·s^−1^ (N·m·kg^−1^)	Pearson’s r	0.35 *	0.35 *	0.33 *
	*p*-value	0.029	0.026	0.037
**(b) Symmetry**				
		**SLH** **(LSI, %)**	**TH** **(LSI, %)**	**COH** **(LSI, %)**
**Proprioception/Kinesthesia**				
JPS 1 (AE_diff_,°)	Pearson’s r	−0.53 *	−0.49 *	−0.70 **
	*p*-value	0.017	0.048	0.001
JPS 2 (AE_diff_,°)	Pearson’s r	−0.46 *	−0.37	−0.44 *
	*p*-value	0.040	0.076	0.050
TTDPM (AE_diff_,°)	Pearson’s r	−0.15	−0.23	−0.21
	*p*-value	0.533	0.278	0.375
**Strength**				
ext 60°·s^−1^ (LSI, %)	Pearson’s r	0.26	0.08	0.13
	*p*-value	0.276	0.423	0.579
flex 60°·s^−1^ (LSI, %)	Pearson’s r	0.18	0.02	0.04
	*p*-value	0.451	0.928	0.864
ext 240°·s^−1^ (LSI, %)	Pearson’s r	0.14	0.15	0.15
	*p*-value	0.545	0.547	0.526
flex 240°·s^−1^ (LSI, %)	Pearson’s r	0.13	0.16	0.13
	*p*-value	0.594	0.268	0.597

Note: JPS: joint position sense; TTDPM: threshold to detection of a passive motion; AE_diff_: between-limb absolute difference; ext: knee extensor muscles; flex: knee flexor muscles; SLH: single hop test; TH: triple hop test; COH: crossover hop test; LSI: limb symmetry index. *p*-value: *: *p* ≤ 0.050; **: *p* ≤ 0.001.

## Data Availability

The data that support the findings of this study are available upon request from the corresponding author, J.D.
